# Multi-Scale Approach for Predicting Fish Species Distributions across Coral Reef Seascapes

**DOI:** 10.1371/journal.pone.0020583

**Published:** 2011-05-26

**Authors:** Simon J. Pittman, Kerry A. Brown

**Affiliations:** 1 Biogeography Branch, Center for Coastal Monitoring and Assessment, National Oceanic and Atmospheric Administration (NOAA), Silver Spring, Maryland, United States of America; 2 Marine Science Center, University of the Virgin Islands, St. Thomas, United States Virgin Islands; 3 School of Geography, Geology, and the Environment, Kingston University London, Kingston-Upon-Thames, United Kingdom; Smithsonian's National Zoological Park, United States of America

## Abstract

Two of the major limitations to effective management of coral reef ecosystems are a lack of information on the spatial distribution of marine species and a paucity of data on the interacting environmental variables that drive distributional patterns. Advances in marine remote sensing, together with the novel integration of landscape ecology and advanced niche modelling techniques provide an unprecedented opportunity to reliably model and map marine species distributions across many kilometres of coral reef ecosystems. We developed a multi-scale approach using three-dimensional seafloor morphology and across-shelf location to predict spatial distributions for five common Caribbean fish species. Seascape topography was quantified from high resolution bathymetry at five spatial scales (5–300 m radii) surrounding fish survey sites. Model performance and map accuracy was assessed for two high performing machine-learning algorithms: Boosted Regression Trees (BRT) and Maximum Entropy Species Distribution Modelling (MaxEnt). The three most important predictors were geographical location across the shelf, followed by a measure of topographic complexity. Predictor contribution differed among species, yet rarely changed across spatial scales. BRT provided ‘outstanding’ model predictions (AUC = >0.9) for three of five fish species. MaxEnt provided ‘outstanding’ model predictions for two of five species, with the remaining three models considered ‘excellent’ (AUC = 0.8–0.9). In contrast, MaxEnt spatial predictions were markedly more accurate (92% map accuracy) than BRT (68% map accuracy). We demonstrate that reliable spatial predictions for a range of key fish species can be achieved by modelling the interaction between the geographical location across the shelf and the topographic heterogeneity of seafloor structure. This multi-scale, analytic approach is an important new cost-effective tool to accurately delineate essential fish habitat and support conservation prioritization in marine protected area design, zoning in marine spatial planning, and ecosystem-based fisheries management.

## Introduction

Rapid progress is being made in the development and implementation of marine management strategies, including marine spatial planning, to balance multiple conservation and resource use objectives [1], [2], [3]. Although a shift towards managing ecosystem patterns and processes is occurring, for example in ecosystem-based management, most strategies still have a focal species component, with directives to manage and monitor specific endangered, threatened, invasive, economically valuable, rare, keystone or indicator species [4], [5], [6]. To be effective, these strategies require spatially accurate ecological information on the geographical distribution of species, as well as an understanding of the key environmental drivers that determine species distributions. Ecologically meaningful decision-making also requires a better understanding of the statistical interactions between environmental drivers and the presence of threshold effects which are rarely modelled explicitly in marine ecology. An ecological threshold is the point at which there is an abrupt change in an ecosystem quality, property or phenomenon, or where small changes in an environmental driver produce large responses [7].

Tropical coral reef ecosystems typically exist as spatial mosaics of interconnected patches of coral reefs, seagrasses, unvegetated sand and mangroves and represent one of the most biologically diverse ecosystems on earth, but are also one of the most vulnerable to environmental change [8]. The highly heterogeneous spatial patterning of patch types, each of which exhibit different structural attributes, result in seascapes with complex seafloor topography at a range of spatial scales. Fish species distributions and diversity patterns are closely associated with structural characteristics, particularly topographic complexity both within a patch type [9] and across the seascape [10], [11]. However, human activity in the coastal zone combined with hurricanes, disease and thermal stress have resulted in broad-scale loss and degradation of biogenic structure created by reef forming scleractinian corals, seagrasses and mangroves [12], [13], [14].

Over the past 20 years, coral reefs of the Caribbean region have experienced a significant decline in coral cover [13] resulting in a ‘flattening’ of the topographic complexity [15]. A concurrent decline in the abundance of a wide range of fish species has also occurred, with greatest declines recorded for herbivorous, invertivorous and carnivorous fish [16]. The decline is likely to have triggered cascading impacts throughout the ecosystem [17], adding fresh impetus to the urgent need to understand broad-scale environmental correlates, such as topographic complexity that influence species distributions across tropical seascapes [17].

Recent research has demonstrated that individual fish species distributions and fish diversity across coral reef ecosystems can be reliably predicted using maps of seafloor structure or bathymetry [18]. It is likely, however, that over broad spatial scales the relationship is more complex with other variables interacting with bathymetry to influence the suitability of habitat for an organism. Few studies examining habitat suitability, however, have considered the potential statistical interaction between physical structure and the relative geographical location across broad spatial scales. A greater understanding of the spatial patterning of species across coral reef ecosystems will provide information on species-environment relationships and spatial proxies for key ecological processes, such as relative grazing or predation intensity or inter-specific competition that can be inferred from maps of fish distributions.

Predicting species distributions in the marine environment is problematic due to limited availability of biological survey data, yet large amounts of marine data are available as species occurrence or presence only data. Presence-only modelling of species distributions is extensively used for terrestrial species and is increasingly being used for global modelling of fish, seabirds and marine mammals [19]. In addition, comparative studies using multiple algorithms have demonstrated that the choice of algorithm can influence both the predictive accuracy and the relative importance of individual predictor variables [20], [21]. Elith et al. [22] compared 16 predictive modelling techniques, including both conventional and machine-learning algorithms using presence-only data for 226 species from six regions. The authors showed that two state-of-the-art machine-learning algorithms, Boosted Regression Trees (BRT), also referred to as Stochastic Gradient Boosted Regression Trees [23], [24], and MaxEnt [25] consistently outperformed other algorithms. Although machine-learning algorithms are becoming more widely applied in terrestrial ecology, few marine applications exist [21], [26], [27]. For marine fish, Knudby et al. [21] showed that machine learning algorithms, particularly tree-based ensembles provided significant increases in performance over more conventional modelling techniques, such as generalised additive models and linear regression.

Both BRT and MaxEnt algorithms have the ability to fit complex functions including interactions between predictor variables and employ strong regularisation techniques, including cross-validation to avoid overfitting. These algorithms have characteristics that make them appropriate to model complex fish-seascape relationships, but have never before been comparatively evaluated for marine species and environments.

We compared and evaluated the performance of two machine-learning algorithms, boosted regression trees (BRT) and maximum entropy modelling (MaxEnt), to model non-linear species-environment relationships for five common fish species associated with topographically complex Caribbean coral reef ecosystems. Environmental data on seafloor structure was acquired from a single remote sensing device, airborne laser altimetry (Light Detection & Ranging or LiDAR), from which derivative spatial predictors were generated to quantify seafloor geomorphology and across-shelf location. Since little was known about the movement patterns of fish species, a single appropriate spatial scale for measuring functionally meaningful seafloor heterogeneity could not be selected *a priori*, instead we used a multi-scale exploratory approach to quantify seascape structure at five spatial scales (5, 25, 50, 100, & 300 metre radii). Although scale-dependency is well demonstrated in marine ecosystems, few species distribution models have incorporated quantitative data on seascape structure across a range of spatial scales. The primary objectives of this study were to: (1) Determine whether the influence of environmental predictors on species' distribution was scale-dependent; (2) evaluate the utility of environmental data from a single remote sensing device combined with metrics for surface morphology to predict and map fish species distributions across a complex coral reef ecosystem; (3) determine which components of remotely sensed seafloor structure contribute most to the species distribution models; (4) identify threshold effects where changes in environmental variables abruptly influence species occurrence; and (5) evaluate the performance of two different machine-learning modelling algorithms for spatial predictions of marine fish distributions.

## Materials and Methods

### Study Area

**Figure 1:**
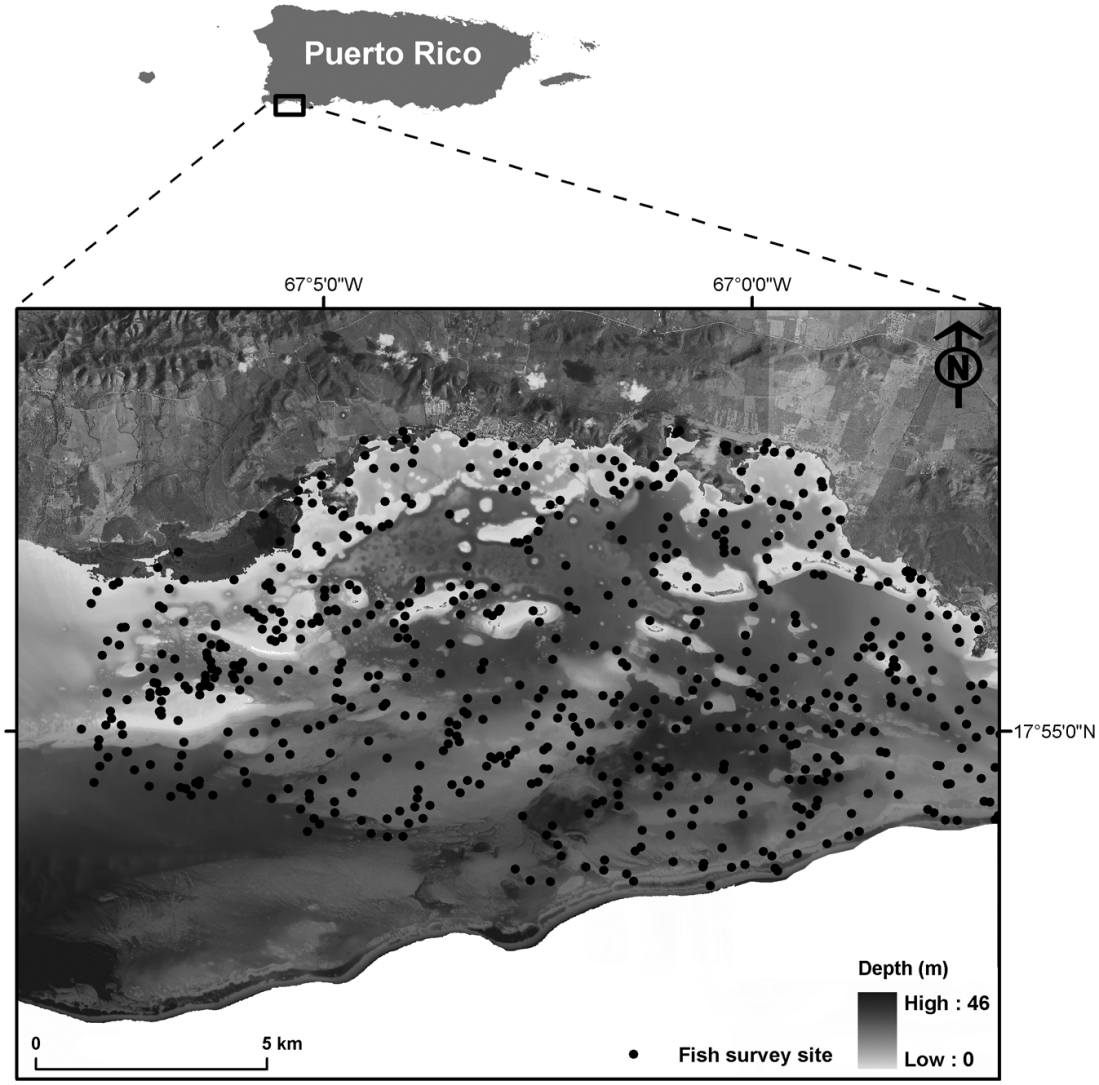
Study area map showing underwater fish survey locations across the La Parguera region of SW Puerto Rico. The underlying data shows the 4 m resolution LiDAR derived bathymetry depicting variability in water depth across the coral reef ecosystems from land to the insular shelf edge in the south.

The coral reef ecosystems of the insular shelf of southwestern Puerto Rico (Fig. 1) exist as a spatial mosaic of habitat types dominated by coral reefs, seagrasses, mangroves and patches of sand. The seafloor is highly heterogeneous in assemblage composition and topographic structure resulting in a diverse and productive fish community, with important ecological, economic and cultural value. In 1979, the La Parguera region (327 km^2^) was designated as a Natural Reserve (NR), Reserva Natural La Parguera, becoming the second marine protected area in Puerto Rico. The La Parguera NR is managed by the Puerto Rican Department of Natural and Environmental Resources (DNER) Bureau of Coastal, Reserves and Refuges (BCRR) as a multiple use zone. Fishing is allowed throughout the Reserve. Like many Caribbean coral reef ecosystems the study area has experienced environmental changes on land and sea that have resulted in loss of structural and functional integrity.

### Fish surveys

Underwater visual surveys of fish and benthic habitat were conducted semi-annually (Jan/Feb and Sept/Oct) across the insular shelf at La Parguera (322 km^2^) between 2001 and 2008. Survey sites (n = 1,018) were selected using a stratified-random sampling design whereby sites were randomly located within two mapped strata (i.e., hardbottom and softbottom) derived from National Oceanic and Atmospheric Administration's nearshore benthic habitat map [28]. The sampling strategy provides a spatially comprehensive and unbiased set of presence records across a wide range of habitat types. Fish surveys were conducted within a 25 m long and 4 m wide (100 m^2^) belt transect deployed along a randomly selected bearing (0–360°). Constant swimming speed was maintained for a fixed duration of fifteen minutes to standardise the search time. All individuals were identified to species level where possible and body lengths (fork length) were visually estimated. To evaluate presence-only modelling algorithms, abundance data for five common species were converted to presence-only data, including (1) coney (*Cephalopholis fulva*) and (2) red hind (*Epinephelus guttatus*) both piscivorous groupers; (3) Princess parrotfish (*Scarus taeniopterus*), an abundant herbivore; (4) Queen triggerfish (*Balistes vetula*), an invertebrate feeder and (5) threespot damselfish (*Stegastes planifrons*), a specialist damselfish, which is known to exhibit a strong positive relationship with several coral species and a preference for topographically complex substrata [29], [30]. The fish species selected for this study minimized the potentially confounding effect of spatial segregation of life stages. For example, juvenile and adult *S. planifrons* and *S. taeniopterus* co-occurred across the study area and *C. fulva*, *E. guttatus* and *B. vetula* were only represented by co-occurring sub-adults and adults. This narrowed the potential niche width and facilitated identification of meaningful environmental predictors. The occurrence varied between species as follows: *C. fulva* (3% of samples); *E. guttatus* (4%), *S. taeniopterus* (23%); *B. vetula* (5%) and *S. planifrons* (6%). Fish data are available online at http://www8.nos.noaa.gov/biogeo_public/query_main.aspx.

### Spatial predictors

Technological advances in sea- air- and space-borne remote sensing devices now provide an unprecedented ability to map the seafloor as a continuously varying three-dimensional surface or bathymetry [31]. New techniques, such as airborne hydrographic LiDAR [32] fire rapid pulses of laser light from an aircraft to the seafloor and sea surface and then measure the difference in the time of reflectance to estimate water depth and hence the vertical height of the seafloor. This technique maps broad areas of shallow water seascapes (<1 m to approx. 50 m) at high spatial resolution (1–16 m^2^).

Bathymetry data were collected for southwestern Puerto Rico between 7th and 15th May 2006 using a LADS (Laser Airborne Depth Sounder) Mk II Airborne System operated by Tenix LADS Incorporated. The laser system was mounted on a DeHavilland Dash 8–200 aircraft flying at survey speeds of 72–90 metres per second and at an altitude of 366–671 metres above the sea surface. A 900 Hertz (1064 nm) Nd:Yaglaser acquired spot data at a rate of 900 pulses per second, with swath widths of 192 metres. This provided post-processing spot data with 4×4 m spacing from <1 m depth to approximately 50 m depth. Erroneous outlying LiDAR returns were removed along with negative values (i.e., land) and mangroves and a seamless bathymetric surface was exported as a GeoTIFF in ArcGIS 9.2 (Environmental Systems Research Institute, Inc.). LiDAR data are available online at http://ccma.nos.noaa.gov/products/biogeography/lidar_pr/welcome.html.

### Quantifying surface morphology

Following Pittman et al. [26], six morphometrics were calculated from the bathymetric surface (mean water depth, aspect, rugosity, slope, slope of the slope and planar curvature i.e. convexities and concavities of the surface) in order to quantify a range of structural attributes from the benthic terrain of southwestern Puerto Rico. To explore the influence of spatial scale on predictive performance, the mean morphometric value of the surrounding seascape was calculated at five spatial scales (5 m, 25 m, 50 m, 100 m and 300 m radius) using a circular moving window within the focal statistics geoprocessing function of ArcGIS's Spatial Analyst (Environmental Systems Research Institute, Inc.). In addition, spatial predictors representing the relative geographical location across the shelf were quantified using a distance to shoreline surface and a distance to shelf edge surface based on Euclidean ‘straight line’ distance. The environmental predictors encompass a comprehensive environmental range from shallow nearshore (<1 m depth) to deeper (max 49 m) shelf edge habitat and from very low relief sandy areas to high relief coral reefs.

### Modelling algorithms

Determination of variable importance and development of predictive models was carried out using Stochastic Gradient Boosting with the Boosted Regression Tree (BRT) code in R software gbm package [33]. BRT is a machine learning algorithm that uses many simple decision trees or ‘ensembles’ to iteratively boost the predictive performance of the final model [24]. Each subsequent regression tree predicts the residuals of the previous thereby learning from the errors or “unsolved cases” of its predecessors. The BRT models were fitted using presence-absence data from 1018 surveyed sites and 11 environmental predictor variables. The model was developed and evaluated using ten-fold cross-validation (CV) to determine the optimal combinations of the learning rate (*lr*) and tree complexity (*tc*), which provided the optimal numbers of trees (*nt*) by minimizing a loss function (i.e., deviance reduction) [34]. *lr* controlled the contribution of each tree to the model using a slow learning rate for all species (0.0001–0.001); while *tc* determined the extent to which statistical interactions were fitted; for instance, a *tc* of two fits a model with two-way interaction. To control for overfitting, BRT uses a regularization process that shrinks individual regression trees, while providing sufficient flexibility to fit complex non-linear relationships. Interaction strength was estimated using the techniques of Elith et al. [30]. The relative contribution of the predictor variables to the final models was determined using the variable importance score based on the improvements of all splits associated with a given variable across all trees in the model, then rescaled so that the most important variable received a score of 100. Other variables received scores that were relative to their contribution to the model's predictive power [34].

Maximum entropy species distribution models were developed with MaxEnt software (MaxEnt v3.3 beta) [25]. MaxEnt relies on presence-only occurrence records to estimate the probability of occurrence for a species, which can then be used to discriminate suitable versus unsuitable areas. MaxEnt finds the probability distribution of maximum entropy (i.e., that is most spread out, or closest to uniform) and then constrains the distribution using a set of environmental variables with a range of values defined by the environment at locations where the species is known to occur [25]. MaxEnt is based on the premise that the unknown probability distribution should have maximum entropy, but is constrained by the environmental characteristics of the niche. MaxEnt controls overfitting and variable selection using a regularisation that smoothes the modelled distribution, with a penalised maximum likelihood model that balances model fit with model complexity [35], [36]. The regularization used by MaxEnt allows it to manage correlated variables [35], which is not the same for the BRT models. However, neither modelling algorithm explicitly treats spatial autocorrelation [37], [38]. Ten-fold cross-validation was used to assess model performance and jackknife resampling to measure the importance of each predictor.

Receiver-operating characteristic curves (ROC) were constructed and the area under the curve (AUC) was used to compare prediction performance [39]. The AUC is a test statistic that uses presence and absence records to assess model predictive performance across a range of thresholds. MaxEnt is a presence-only algorithm; therefore we used the Phillips et al. [25] approach that applied randomly selected pseudo-absences instead of observed absences to ROC AUC. We adopted the interpretation offered by Hosmer and Lemeshow [40] whereby an AUC value of 0.7–0.8 is considered an acceptable prediction; 0.8–0.9 is ‘excellent’ and >0.9 is ‘outstanding’. A value of 0.5 is defined as the predictive ability that could be achieved by chance alone.

Map accuracy was calculated using an independent set of underwater survey data (n = 360) collected using an identical technique to the original survey data used to build the models. Predicted probability of presence sometimes referred to as habitat suitability values were mapped to the 4×4 m cells of the predictors, with values scaled between 0 (absence) and 100 (highest probability of presence). Mapped predictions were converted to binary values (>10% probability = suitable habitat; <10% = unsuitable) and quantitatively assessed. Map accuracy was calculated as the percentage of actual species sightings predicted correctly by the predictive map.

We used generalized linear mixed models (GLMM) to analyse variation in AUC, since the grouping structure for the data consisted of modelling technique (i.e., BRT and MaxEnt), which varied between five species at five different spatial scales. AUC was included as the response variable (with Poisson error distribution) and modelling technique was fitted as a fixed effect. Spatial scale and species were fitted as random factors and an interaction between species and scale was fitted as random factor. Models were evaluated by model selection and likelihood ratio test (LRT). The GLMM was developed using the *glmer* function of the lme4 library in the statistical software package R ver. 2.8.1 [41]. Additionally, simple linear regression was used to examine relationships between fish body length and the spatial scale of seascape structure that contributed most to models.

## Results

### Comparison of BRT and MaxEnt models

BRT provided ‘outstanding’ model predictions (AUC = >0.9) for three of five species and the remaining two considered ‘excellent’ and ‘acceptable’. MaxEnt provided ‘outstanding’ model predictions for two out of five species with the remaining three models considered ‘excellent’ (AUC = 0.8–0.9) according to the criteria of Hosmer and Lemeshow [40] (Table 1). At the species level, BRT and MaxEnt models for *C. fulva* performed best (BRT AUC = 0.97; MaxEnt AUC = 0.94) followed by BRT models for *S. taeniopterus and S. planifrons* (AUC = 0.93 and 0.92 respectively). The lowest performing was a BRT model for *E. guttatus* (AUC = 0.74) and MaxEnt models for *S. taeniopterus* (mean AUC = 0.84).

**Table 1:** Cross-validation AUC values from BRT and MaxEnt with best performing models for each algorithm highlighted in bold.

Species	Scale (m)	BRT AUC	MaxEnt AUC
*B. vetula*	5	0.846	0.861
*B. vetula*	25	0.831	0.855
*B. vetula*	50	0.838	0.854
*B. vetula*	100	0.852	0.858
*B. vetula*	300	**0.867**	**0.862**
Mean SE		0.847 (0.02)	0.858 (0.002)
*C. fulva*	5	0.952	0.833
*C. fulva*	25	0.972	0.936
*C. fulva*	50	0.970	**0.940**
*C. fulva*	100	**0.973**	0.937
*C. fulva*	300	0.962	0.939
Mean SE		0.966 (0.01)	0.917 (0.02)
*E. guttatus*	5	0.771	**0.862**
*E. guttatus*	25	**0.774**	0.848
*E. guttatus*	50	0.749	0.854
*E. guttatus*	100	0.759	0.848
*E. guttatus*	300	0.77	0.847
Mean SE		0.765 (0.02)	0.851 (0.003)
*S. planifrons*	5	0.916	0.886
*S. planifrons*	25	**0.925**	0.892
*S. planifrons*	50	0.920	0.900
*S. planifrons*	100	0.908	**0.901**
*S. planifrons*	300	0.894	0.891
Mean SE		0.913 (0.01)	0.894 (0.003)
*S. taeniopterus*	5	0.911	0.819
*S. taeniopterus*	25	0.928	0.834
*S. taeniopterus*	50	**0.932**	0.848
*S. taeniopterus*	100	0.931	0.848
*S. taeniopterus*	300	0.928	**0.851**
Mean SE		0.926 (0.009)	0.840 (0.006)
Total Model Mean		0.883	0.872

The models are for *Balistes vetula* (Queen triggerfish), *Cephalopholis fulva* (coney), *Epinephelus guttatus* (red hind), *Stegastes planifrons* (threespot damselfish) and *Scarus taeniopterus* (Princess parrotfish). The highest AUC for each modelling technique is shown in bold.

The GLMM analysis showed that there was no significant effect of AUC on modelling technique (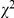 = 0.001; *p*>0.05; LRT); there was also no effect of scale (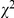 = 0.002; P>0.05; LRT), species (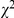 = 0.116; *p*>0.05; LRT), nor interaction between scale and species (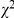 = 0.118; *p*>0.05; LRT) on model performance. Additionally, model performance was not significantly (p = >0.05) correlated with species prevalence for BRT or MaxEnt models (r^2^ = 0.07 and 0.24 respectively). For ease of presentation, the following results focus on the best BRT models (Table 2).

**Table 2:** Optimal settings and predictive performance for Boosted Regression Tree models.

Species	Scale (m)	No. trees	Learn rate	tc	CV Deviance	SE
*B. vetula*	5	4150	0.0005	5	0.33	0.014
*B. vetula*	25	2250	0.0009	5	0.326	0.015
*B. vetula*	50	2800	0.0008	5	0.326	0.015
*B. vetula*	100	3700	0.0008	4	0.321	0.020
*B. vetula*	300	6250	0.0004	5	0.308	0.017
*C. fulva*	5	3000	0.001	3	0.162	0.021
*C. fulva*	25	3700	0.0009	5	0.163	0.016
*C. fulva*	50	6900	0.0004	5	0.164	0.013
*C. fulva*	100	5500	0.0006	5	0.158	0.017
*C. fulva*	300	4700	0.0006	5	0.158	0.015
*E. guttatus*	5	3500	0.0003	4	0.297	0.008
*E. guttatus*	25	3400	0.0003	5	0.296	0.007
*E. guttatus*	50	4450	0.0002	5	0.301	0.008
*E. guttatus*	100	8350	0.0001	5	0.297	0.005
*E. guttatus*	300	9050	0.0001	5	0.293	0.008
*S. planifrons*	5	8050	0.0009	4	0.502	0.029
*S. planifrons*	25	8950	0.0007	5	0.461	0.015
*S. planifrons*	50	7450	0.0007	5	0.478	0.039
*S. planifrons*	100	6350	0.0007	4	0.509	0.040
*S. planifrons*	300	7700	0.0006	5	0.548	0.028
*S. taeniopterus*	5	4800	0.0009	5	0.592	0.026
*S. taeniopterus*	25	4750	0.0009	4	0.567	0.038
*S. taeniopterus*	50	6050	0.0008	4	0.549	0.021
*S. taeniopterus*	100	5650	0.0008	4	0.555	0.028
*S. taeniopterus*	300	6200	0.0008	4	0.552	0.029

The models are for *Balistes vetula* (Queen triggerfish), *Cephalopholis fulva* (coney), *Epinephelus guttatus* (red hind), *Stegastes planifrons* (threespot damselfish) and *Scarus taeniopterus* (Princess parrotfish). The bag fraction is 0.50 for all models unless indicated differently.

### Variable contributions and threshold effects

**Figure 2:**
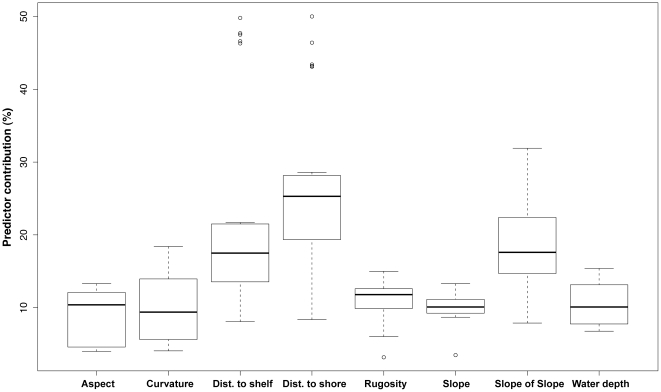
Boxplots of percentage contribution of each environmental predictor across all models and spatial scales for five fish species. Horizontal lines in boxes show medians and boxes show upper and lower quartiles, with vertical lines showing minimum and maximum values. Distance is abbreviated “Dist”.

**Figure 3:**
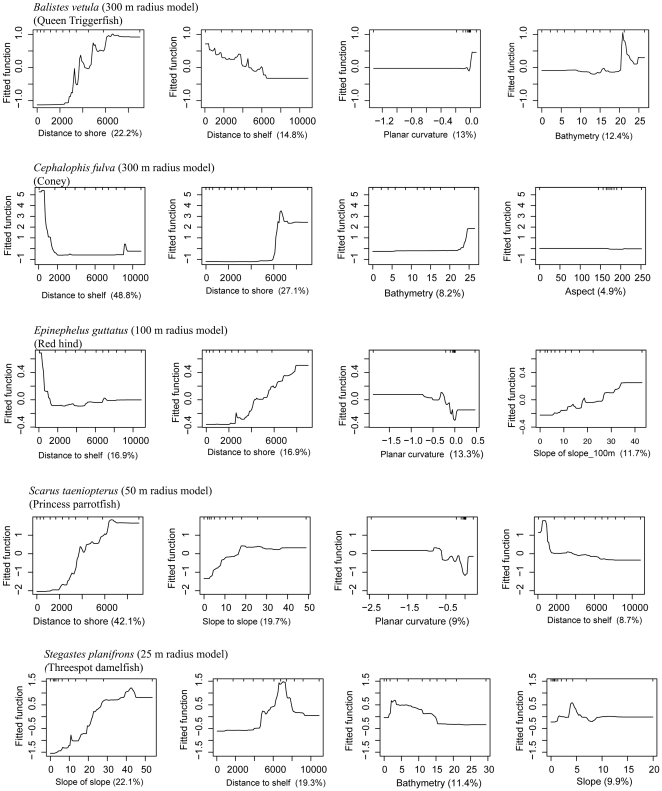
Partial dependence plots for Boosted Regression Tree (BRT) analyses relating species occurrence to the top 4 most influential geographical and morphological predictors. (A) *Balistes vetula* (Queen triggerfish); (B) *Cephalopholis fulva* (coney), (C) *Epinephelus guttatus* (red hind); (D) *Scarus taeniopterus* (Princess parrotfish); and (E) *Stegastes planifrons* (threespot damselfish). The graphs show the effect of a particular variable on the response: positive fitted function values suggest that species respond favorably and low values suggest the opposite. The relative importance of each variable is shown in parentheses on the *x*-axis. Increasing negative values for planar curvature represent increasing amount of convexity in the surface; positive values are concavity.

Our findings revealed that the single most influential predictor was geographical location across the shelf, represented by distance to the shelf edge and distance to the shoreline (Fig. 2). Distance to shelf was the primary predictor for the two grouper species and distance to shore for the Princess parrotfish and Queen triggerfish. *B. vetula*, *C. fulva* and *E. guttatus* exhibited similar predictor relationships: whereby species occurrence was predicted to be higher in seascapes that were farthest offshore (Fig. 3). For *C. fulva*, a threshold effect was evident at approximately 2000 metres from the shelf edge, where species occurrence abruptly increased (Fig. 3). *C. fulva* and *B. vetula* responded positively to areas with greater depths (20–25 m). *S. taeniopterus* also showed a preference for offshore habitat, but with a more gradual pattern of increasing occurrence predicted across the shelf beyond 2000 meters from shore (Fig. 3).

**Figure 4:**
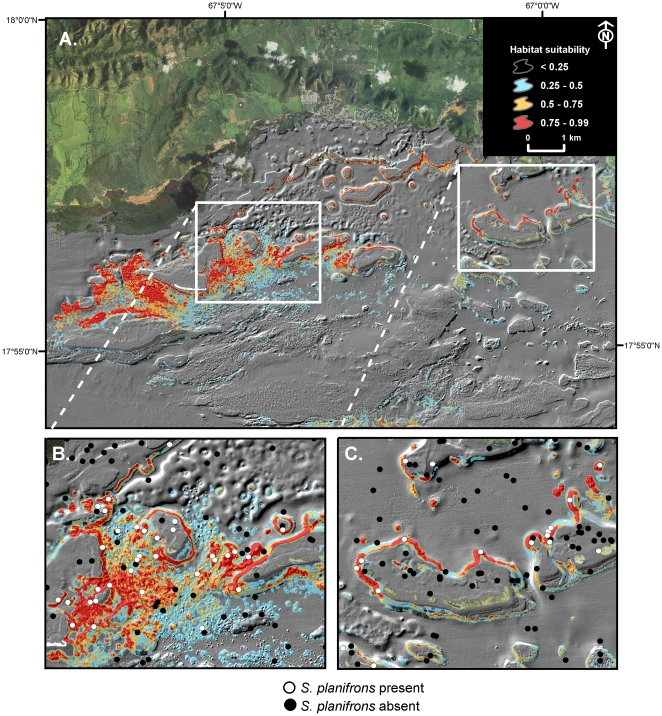
Predicted habitat suitability for *Stegastes planifrons* for the study area of SW Puerto Rico. (A) MaxEnt model of habitat suitability for *S. planifrons* overlain on 4 m resolution LiDAR bathymetry; (B) Subset of the habitat suitability map for *S. planifrons* showing a high density of highly suitable habitat (red) around the El Palo reef area within the La Parguera Natural Reserve; and (C) Subset of the habitat suitability map showing highly suitable habitat (red) predicted along the shallow landward reef slopes near Corral and Romero cays. Sites of confirmed presence and absence of *S. planifrons* are represented by white and black dots respectively.

Of the morphometrics, topographic complexity (i.e., slope of slope) was most influential in determining occurrence of *S. planifrons*, *S. taeniopterus* and *E. guttatus*. Slope of slope was the primary predictor for *S. planifrons* with a strong interaction with distance to the shelf edge. More specifically, *S. planifrons* occurrence was predicted to be highest in high complexity areas between 4,000 m and 7,500 m from the shelf edge and in depths shallower than 15 m. The spatial prediction of probability of presence or habitat suitability revealed a high density of highly suitability habitat for *S. planifrons* over shallow water aggregated patch reefs with high topographic complexity and along the landward slopes of shallow linear reefs fringing offshore cays and emergent reefs (Fig. 4). The two relationships that contributed most to regulating the distribution of *S. taeniopterus* were proximity to shore (negative relationship) and slope of slope (positive relationship), suggesting that both geographic and topographic variables are also important for this species.

The strength of the response curve and the location of the point at which increasing topographic complexity no longer led to increasing occurrence differed between species. For *S. taeniopterus*, a gradual increase in occurrence with increasing slope of slope was predicted with even very small increases in complexity greater than zero (flat bottom) resulting in occurrence. Complexity increased habitat suitability until slope of slope values reached approximately 20, beyond which habitat suitability levelled off. A steeper response curve was evident for *S. planifrons*, with occurrence increasing with complexity up to a slope of slope value of 45 (Fig. 3). *E. guttatus* increased gradually with slope of slope until a value of approximately 35, where a plateau in the response occurred. These findings highlight the existence of species-specific responses to topographic complexity, as well as some generality in the importance of the interaction between geographical location across the shelf and topographic complexity of the seascape for predicting fish distributions across coral reef ecosystems.

### Variable interactions

**Figure 5:**
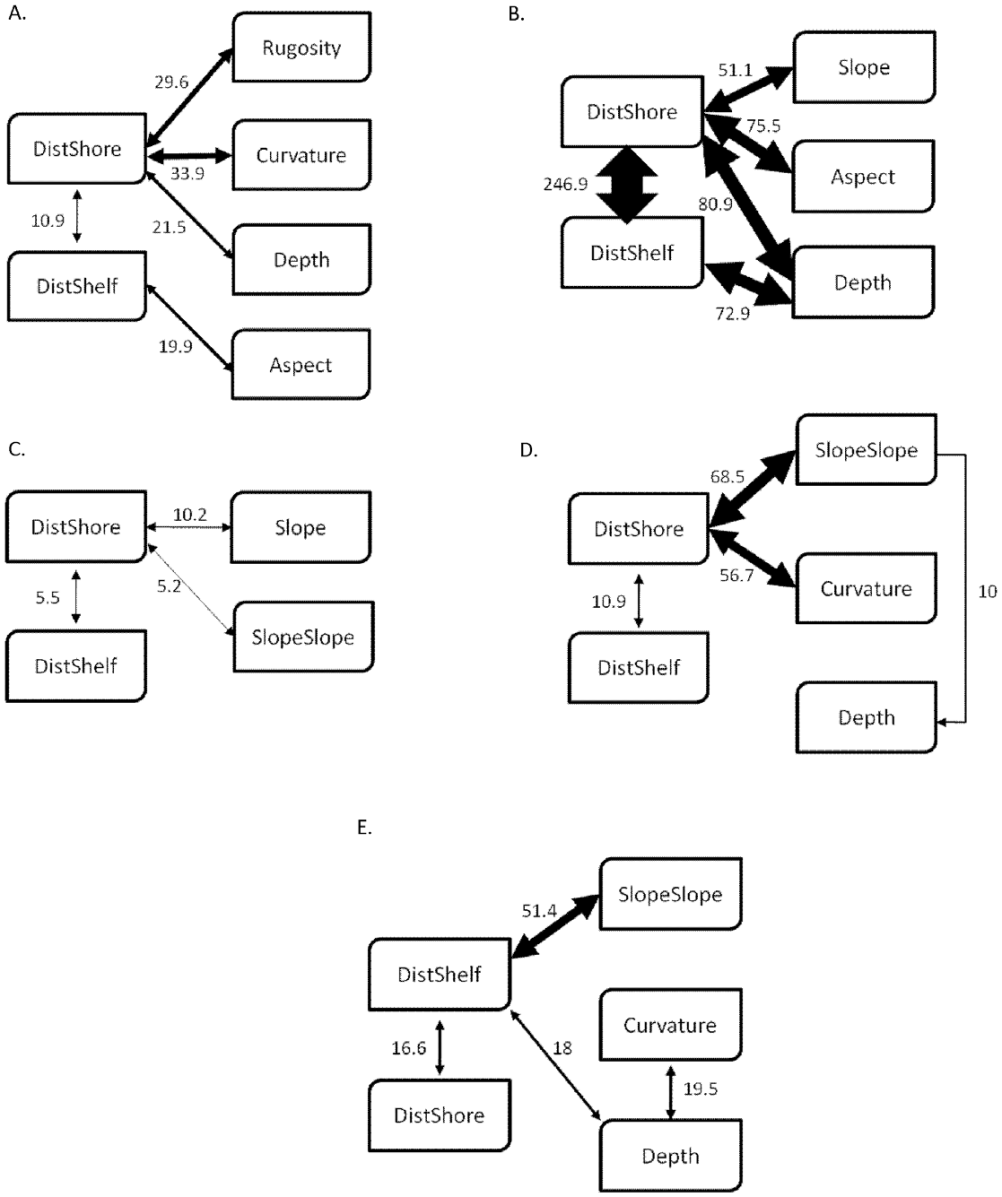
Schematic models of predictor interactions from BRT models. (A) *Balistes vetula* (Queen triggerfish); (B) *Cephalopholis fulva* (coney); (C) *Epinephelus guttatus* (red hind); (D) *Scarus taeniopterus* (Princess parrotfish); (E) *Stegastes planifrons* (threespot damselfish). Line thickness is proportional to interaction strength with thicker lines indicating stronger interactions.

Although variable importance did not fluctuate across spatial scales for the fish species investigated, the interactions between topographic and geographic predictors led to a more ecologically meaningful understanding of how multiple predictors interact to determine habitat suitability. This was particularly true for *B. vetula*, *E. guttatus*, *S. planifrons* and *S. taeniopterus*. For instance, the most important interactions for *S. planifrons* were consistently between the slope of slope and distance to shore (Fig. 5). A similar result was exhibited for *E. guttatus*, *S. planifrons* and *S. taeniopterus*. Aside from the expected interaction between the inversely related distance to shore and distance to shelf edge, interaction strength was highest for: i.) *B. vetula* – distance to shore and curvature and rugosity; ii.) *C. fulva* - distance to shore and water depth; iii.) *E. guttatus* - distance to shore and slope; iv.) *S. taeniopterus* - distance to shore and slope of slope; and v.) *S. planifrons* - distance to shelf edge and slope of slope. The model for *C. fulva* involved the strongest interactions among predictors. In contrast, *E. guttatus* exhibited relatively weak interactions. Moreover, the most important interactions for *B. vetula and E. guttatus* tended to vary across spatial scales, suggesting that the synergistic effects of different predictors are important for regulating species' distribution across scales in Caribbean coral reef seascapes.

### Influential spatial scales

The best BRT models for *B. vetula* and *S. taeniopterus* were developed using environmental predictors at the 300 m scale. The best model for *C. fulva* was at the 100 m scale; *S. planifrons* at the 25 m scale; and *E. guttatus* at the 5 m scale (data not shown). Although the strength of the response varied across spatial scales, rarely did the relative importance of different environmental variables change across spatial scales for any species' models since the primary and secondary predictors (i.e., the Euclidean distance from shelf edge and shore) were scale-independent metrics.

### Map accuracy of predicted species distributions

Independent map accuracy assessment demonstrated that MaxEnt models produced more reliable spatial predictions of species occurrence than did BRT models (Table 3). Map accuracy for MaxEnt models was consistently high across all five species, with highest accuracy calculated for *S. taeniopterus* (97% correct) and *C. fulva* (95.8% correct). Predictive maps projected from BRT models were less reliable for all species than MaxEnt and more variable, ranging from 48% accuracy for *B. vetula* to 96.6% for *S. taeniopterus* (Table 3).

**Table 3:** Comparison of map accuracy for predicted fish species distributions using BRT and MaxEnt algorithms.

Species	Presence sites	BRT		MaxEnt	
		% Correct	% Misclassified	% Correct	% Misclassified
*B. vetula*	44	48	52	90	10
*C. fulva*	24	54.2	45.8	95.8	4.2
*E. guttatus*	10	70	30	90	10
*S. planifrons*	30	73.3	26.7	90	10
*S. taeniopterus*	87	96.6	3.4	97	3
**Mean**		**68.4**	**31.6**	**92.6**	**7.4**

Prediction probability threshold of >10% used for mapping suitable habitat. Mean values are bold.

## Discussion

The spatial modelling approach developed here integrates data and novel tools and techniques from geographical information science together with landscape ecology concepts and advanced machine-learning algorithms to model complex non-linear species-environment relationships. We have demonstrated that morphological characteristics of the seafloor and geographical predictors interact to function as effective predictors of fish species distribution across topographically complex coral reef ecosystems. Our results demonstrated that coral reef ecosystems exhibit high spatial variability in habitat suitability at a range of scales for five common fish species. We demonstrate that the location of coral reefs across the insular shelf does matter to fish; and that coral reefs of equally high topographic complexity will not necessarily offer identical habitat suitability for fish.

Although species showed individualistic responses to predictors, non-linear statistical interactions between the geographical location across the shelf and the structural heterogeneity of the seafloor produced reliable models of species distributions. Geographical threshold effects were evident in ecological responses for several species indicative of distinct zonation in the spatial pattern of habitat suitability. The ability to map the spatial patterns in habitat quality for species and groups of species is valuable for mapping essential fish habitat and conservation planning.

Most importantly, we highlight the importance of using independent validation data to evaluate model predictions and demonstrate that model performance may not necessary translate to map accuracy when the predictions of habitat suitability are projected across seascapes. Higher map accuracy from MaxEnt model predictions may reflect the difference between an entropic distribution with environmental constraints capable of modelling very complex spatial distributions, versus a recursive partitioning approach with splitting across variable values. Splitting may perform better for species with more distinct zonation patterns of distribution. More multi-species studies are required to examine the distributional characteristics that specific algorithms are best suited to predict. Moreover, rather than relying solely on AUC, alternative metrics should be used to evaluate model performance, particularly for MaxEnt which relies on pseudo-absences. Using pseudo-absences may lead to biased AUC values, because this index gives equal weights to omission and commission errors and pseudo-absences tend to inflate the number of false absences [42]. However, for our data, using independent validation data to assess model predictions favoured MaxEnt.

### Variable contribution and interactions

Topographic complexity is widely recognised as an important predictor of fish species distributions, with more complex patches and seascapes supporting higher fish abundance and species richness than less complex patches [10], [11], [21], [43]. Although our results support this hypothesis, with slope of the slope and surface rugosity (measures of topographic complexity) identified as important predictors in distribution models of three of the five fish species, we also show that not all coral reefs offer equal habitat suitability, even if they do exhibit equal levels of topographic complexity. At broad spatial scales, the suitability of coral reefs for fish species in the study area was mediated by the interaction between topographic complexity and geographical location across the insular shelf. In fact, cross-shelf location measured by Euclidean distance from both the shelf edge and shoreline explained more of the variability in fish species occurrence than any other individual predictor.

Several studies have highlighted the importance of cross-shelf location for fish distributions [44], [45], [46], yet relative position across the shelf is rarely directly quantified as a potential spatial proxy in ecological studies of marine species distributions. Both distance to coastline and distance to barrier reef emerged as the most important predictors for a wide range of fish species on the Great Barrier Reef, Queensland Australia [47], [48]. A disadvantage associated with use of a geographical predictor is that the exact causal patterns and processes relevant to cross-shelf location are ambiguous. An advantage, however, is that geographical predictors provide a relatively static, easy to quantify proxy that may indirectly represent changes across a wide range of dynamic gradients in environmental conditions (e.g., depth, temperature, salinity, turbidity, connectivity) including those that are problematic to quantify accurately at appropriate spatial and temporal scales.

Compared with geographical predictors and topographic complexity, other predictors such as curvature, aspect and slope each contributed less than 12% (mean variable contribution) across all species. These variables have been found to be important predictors of vegetation distribution in terrestrial landscapes, yet very little is known about their importance as drivers of ecological patterns across the seascape. Slope and aspect could influence hydrodynamics and the amount of light irradiance received by photosynthetic organisms (e.g., algae and scleractinian corals), with implications for fish distributions; but these characteristics of the terrain morphology have yet to be explored relative to biological function in coral reef ecosystems.

### Threshold effects

This study identified several thresholds in predictor responses to geographical location, which defined discrete constraints on habitat suitability across the shelf. This pattern is indicative of the existence of ecologically meaningful zonation across the shelf likely mediated by local coastal geomorphology. The existence of geographical threshold effects may be related to life-history strategies and tactics, such as whether a species is a habitat specialist with a critical dependence on a single habitat type or seascape generalist capable of using multiple habitat types and geomorphological zones. Evidence from terrestrial species [49] and a few marine examples [26], [50] indicate that threshold effects are species specific, a result that was supported by our findings. Past studies have focused on changes or spatial differences in the abundance of patch types represented as two dimensional flat surfaces, rather than spatial gradients of three dimensional surfaces as was accomplished with these analyses. Our study suggests that understanding the three dimensional structural conditions under which thresholds are likely to be exceeded and the mechanisms underlying the threshold response is critical to predicting change and for examining the options for management intervention and setting targets for structural restoration.

### Spatial scale

Our multiscale approach, adapted from landscape ecology, allowed us to examine scale-dependent effects in species response to environmental heterogeneity. A range of spatial scales emerged for identifying the characteristic scale of response for the five fish species. The scale of response was species specific with no positive allometric scaling relationship evident between fish body size and size of seascapes. For instance, one of the grouper species (*E. guttatus*) was best predicted using spatial complexity quantified at the 5 m radial extent, while the smallest bodied fish species (*S. planifrons*) was best predicted at the 25 m radial extent. This may, however, reflect a site specific preference for highly complex structure in close proximity for *E. guttatus*. Limited information is available on the scale of movements for most tropical species, therefore limiting any meaningful scale selection in ecology studies. Where data are available, behavioural studies have shown that *S. planifrons* is a highly territorial site-attached fish with a home range of several metres and no evidence for nocturnal migrations [51]. In contrast, behavioural observations of *S. taeniopterus* in Barbados found that fish moved (20 to 375 m migrations) to structurally complex and deeper reef slopes or nearby areas with high coral colony density to find night resting areas [52]. It is likely that suitable habitat includes close proximity between day and night use areas that together offer sufficient structural complexity to provide abundant food and refuge from predators. The proximity of suitable habitats determines both the spatial scales of the daily home range and therefore the spatial scales at which individuals respond to the environment. For species such as *S. taeniopterus*, an exploratory, multi-scale approach that is inclusive of surrounding structural heterogeneity at a range of scales is more likely to include the structurally complex night resting areas that exist within a few hundred metres of the locations of daytime occurrence.

### Management implications and future challenges

A systematic assessment of marine species' distributions and their responses to specific environmental variables at multiple spatial scales provides valuable information for conservation planning and fisheries management. The quantitative and spatially explicit techniques demonstrated offer a cost-effective and reliable tool for refining the spatial delineation of essential fish habitat within a region and for identifying the suite of site characteristics that are important for priority species [53]. Furthermore, a multi-scale approach obviates some of the minor geopositional inaccuracies that may occur in field surveys when linking response variables to environmental structure. A multi-scale approach is ecologically appropriate when insufficient information on actual movements and habitat use patterns are available, and when it is likely that species respond hierarchically to spatial structure and respond at different scales to different components of structure [50], [54].

Future studies are now underway that examine the potential applications of our modelling approach for forecasting the influence of differing levels of ‘topographic flattening’ on habitat suitability and the associated contractions and expansions in fish species distribution. Species distributions can also be used as spatial proxies for key ecological processes such as herbivory. Mapped distributions for multiple herbivorous species can be spatially combined to map cumulative patterns of grazing intensity, a key process controlling the dynamics and resilience of coral reef ecosystems [55].

Additional future work is required to determine the portability and generality of the models through application to geographically different regions and to assess performance for a wider range of species in both fished and unfished areas [56]. Spatial modelling techniques can offer a cost-effective analytical solution to both filling the spatial information gap and increasing our understanding of macro-ecological relationships, even in relatively data poor regions of the world. The results emphasise the importance of understanding the architectural complexity of coral reefs, particularly in the Caribbean and other sensitive seascapes that have shown declines in coral cover and a ‘flattening’ of coral topography as a result of catastrophic and sub-catastrophic events including disease, hurricanes and bleaching.
